# Synthesis and Photocatalytic Evaluation of CoPc/g-C_3_N_4_ and CuPc/g-C_3_N_4_ Catalysts for Efficient Degradation of Chlorinated Phenols

**DOI:** 10.3390/molecules31020213

**Published:** 2026-01-08

**Authors:** Cagla Akkol, Yasemin Caglar, Ece Tugba Saka

**Affiliations:** 1 Department of Chemistry, Faculty of Science, Karadeniz Technical University, Trabzon 61080, Türkiye; akkolcagla@gmail.com; 2 Department of Chemistry, Institue of Science, Karadeniz Technical University, Trabzon 61080, Türkiye; 3 Department of Genetic and Bioengineering, Faculty of Engineering, Giresun University, Giresun 28200, Türkiye

**Keywords:** phthalocyanine, graphitic carbon nitride, phenol degradation, photocatalysis

## Abstract

The oxidation of chlorophenolic compounds is essential for converting these persistent and toxic pollutants into less harmful products, thereby reducing their environmental and health impacts. In this study, a p-coumaric acid ester derivative was employed as the starting material to synthesize the corresponding phthalonitrile precursor (EnCA-CN), followed by the preparation of non-peripherally substituted Co(II) and Cu(II) phthalocyanine complexes (EnCA-Copc and EnCA-CuPc). These complexes were subsequently characterized using a range of spectroscopic techniques and designed to engage in π–π interactions with graphitic carbon nitride to form efficient photocatalytic materials. The structures of the two effective catalysts were characterized by FT-IR, SEM, and XRD analyses, after which their photocatalytic performance and recyclability in the degradation of 2-chlorophenol, 2,3-dichlorophenol, and 2,3,6-trimethylphenol were evaluated. The optimum catalyst loading for the MPc/g-C_3_N_4_ composites was determined to be 0.5 g/L, yielding the highest photocatalytic efficiency. The EnCA-CoPc/g-C_3_N_4_ catalyst achieved 90.8% product selectivity and 82.6% conversion in the oxidation of 2-chlorophenol, whereas the EnCA-CuPc/g-C_3_N_4_ catalyst exhibited approximately 80.0% pollutant removal. The degradation efficiencies followed the order 2-CP > 2,3-DCP > 2,3,6-TCP, with benzoquinone derivatives identified as the major oxidation products. In recyclability tests, both catalysts retained more than 50% of their activity after five cycles; EnCA-CoPc/g-C_3_N_4_ maintained 68% conversion in the 5th cycle, while EnCA-CuPc/g-C_3_N_4_ retained 60% conversion in the 4th cycle.

## 1. Introduction

Chlorophenol derivatives are recognized as significant environmental pollutants due to their high toxicity, persistence, and tendency to bioaccumulate within ecosystems. Their long-term stability in aquatic and soil environments poses risks to ecological balance and human health. Therefore, the effective degradation and removal of chlorophenols is crucial for sustainable environmental management and pollution control [1,2,3,4]. Therefore, the oxidation reactions of chlorophenols are of particular importance, as they facilitate their degradation and conversion into less harmful species. In this context, the development and application of new catalytic systems for oxidation processes have gained increasing attention, with the aim of achieving more environmentally sustainable approaches through reduced time and energy consumption [5,6,7]. Phthalocyanines—particularly metallophthalocyanines (MPc)—exhibit intense and broad absorption in the visible-light region, efficient photoinduced charge generation and transfer, and an extended π-conjugated framework. In addition, MPc complexes contain redox-active metal centers that can effectively participate in electron-accepting or electron-donating pathways [8,9,10]. When integrated with g-C_3_N_4_, these properties collectively broaden the light-harvesting range toward longer red wavelengths and promote interfacial charge separation either from g-C_3_N_4_ to MPc or vice versa, thereby suppressing electron–hole recombination [11,12,13]. Moreover, the tunability of the metal center (e.g., Co, Fe, Cu) introduces catalytic sites that enhance substrate activation and overall photocatalytic turnover. The synergistic electronic coupling and π–π interactions between MPc and g-C_3_N_4_ further stabilize charge carriers, resulting in significantly improved photocatalytic efficiency in oxidation and degradation processes [14,15,16].

A photocatalyst is a semiconductor material capable of initiating redox reactions under light irradiation. Upon illumination, photoexcited charge carriers generate oxidative and reductive active sites on the surface, enabling electron-transfer processes [17,18]. Electrical conductivity arises when electrons are promoted from the valence band to the conduction band, and the relatively narrow bandgap of semiconductors allows for efficient charge migration between these bands [19,20]. External stimuli such as light, heat, or an applied electric field can induce these transitions. Additionally, factors such as charge-carrier lifetime, crystallinity, and heterojunction formation significantly influence charge separation efficiency and, consequently, the overall photocatalytic performance. In this study, Co(II) and Cu(II) phthalocyanine complexes non-peripherally substituted with ethyl p-coumarate groups were synthesized and characterized in the initial stage. Ethyl p-coumarate is well recognized for its strong antioxidant, antimicrobial, antifungal, and antiradical activities [21]. The synthesis of EnCA-substituted phthalocyanine compounds (EnCA-CoPc and EnCA-CuPc) bearing paramagnetic metal centers was undertaken with the aim of developing new photocatalysts capable of degrading synthetic phenolic pollutants under light irradiation. By integrating the redox-active catalytic properties of metal-centered phthalocyanines with the broad light absorption characteristics of graphitic carbon nitride, the optimal conditions for the photocatalytic degradation of chlorophenols were established.

## 2. Results and Discussion

### 2.1. Synthesis and Characterization

**Figure 1:**
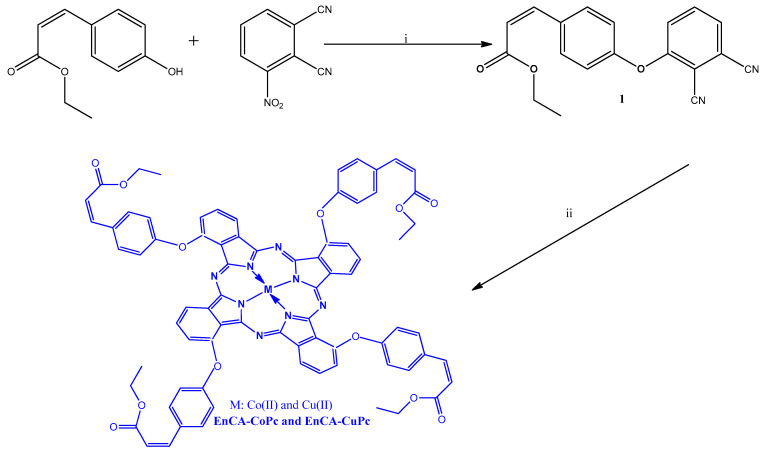
All synthetic pathways to synthesis the molecules. (i: K_2_CO_3_, DMF, 50 °C, 72 h, ii: n-pentanol, DBU, CoCl_2_ or CuCl_2_, 160 °C, 24 h).

**Figure 2:**
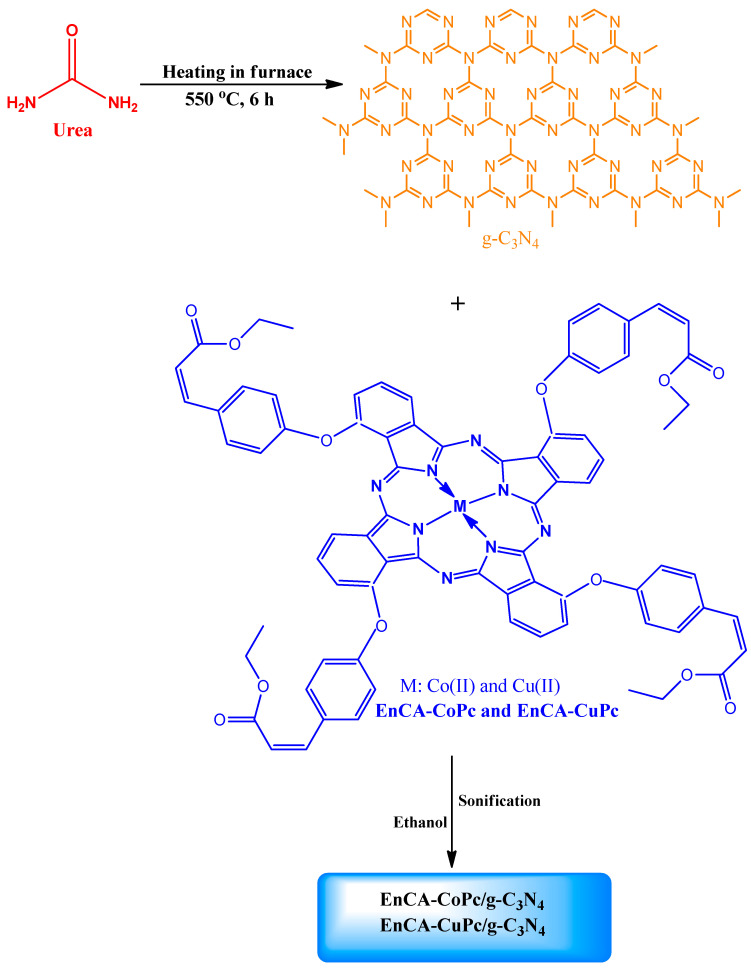
The reaction routes for preparation of metallophthalocyanine/graphitic carbon nitride structures.

**Figure 3:**
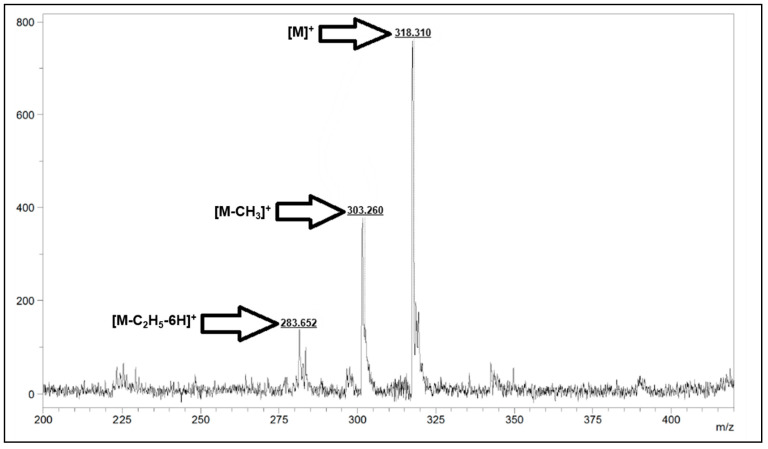
MALDI-TOF spectrum of ethyl (2Z)-3-(4-(2,3-dicyanophenoxy)phenyl)acrylate (EnCA-CN).

All synthetic pathways were given in Figure 1 and Figure 2. EnCA-CN was successfully synthesized and its structure was verified by FT-IR, NMR and Mass spectral techniques. In the FT-IR spectrum, the sharp absorption at 2225 cm^−1^ corresponds to the C≡N stretching vibration of the dicyano group, while the strong band at 1703 cm^−1^ confirms the ester C=O group. The characteristic aromatic (3078 cm^−1^) and aliphatic (2987 cm^−1^) C–H vibrations were also evident (Supplementary Figure S1). The ^1^H-NMR spectrum exhibited multiple aromatic proton signals between 7.52–8.13 ppm, confirming the presence of phenyl and phenoxy moieties. The signal at 6.62 ppm is attributed to the CH=CH protons of the conjugated acrylate group, the quartet at 4.17–4.23 ppm (CH_2_–O) and the triplet at 1.25 ppm (CH_3_) correspond to the ethyl ester moiety (Supplementary Figure S2). In ^13^C-NMR spectrum, the resonance at 166.62 ppm is assigned to the ester carbonyl carbon, while the signals between 115–123 ppm correspond to the cyano carbons (Supplementary Figure S3). MALDI-TOF-MS analysis showed a molecular ion peak at *m*/*z* = 318.310 [M]^+^, consistent with the calculated value (318.32 g/mol), confirming the successful formation of EnCA-CN (Figure 3).

**Figure 4:**
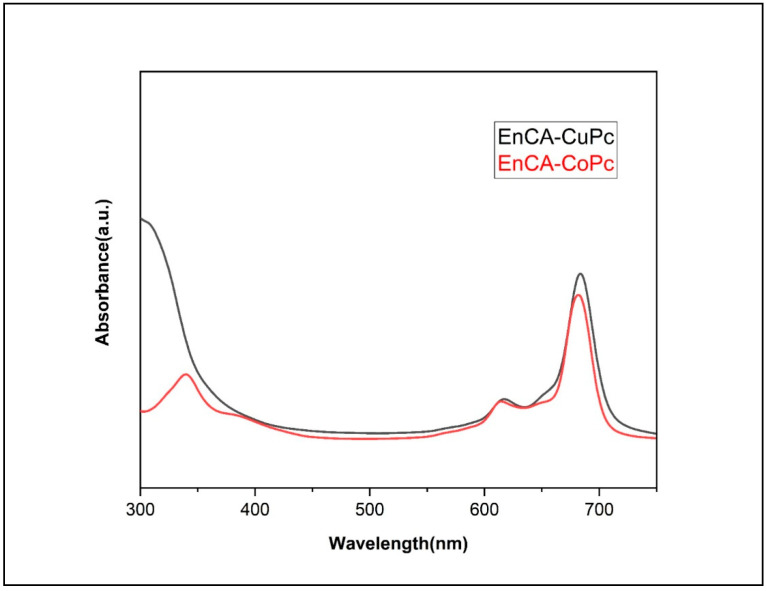
UV-Vis spectrum of EnCA-CoPc and EnCA-CuPc.

**Figure 5:**
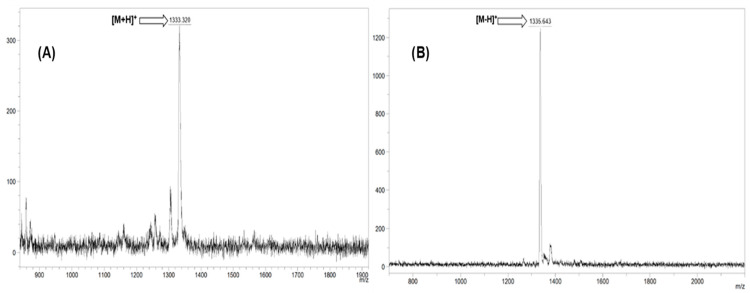
MALDI-TOF spectra of (**A**) EnCA-CoPc and (**B**) EnCA-CuPc.

FT-IR spectrum revealed the retention of the C=O stretching at 1705 cm^−1^ for EnCA-CoPc and 1712 cm^−1^ for EnCA-CuPc. The aromatic C–O stretching bands at around 1250–1174 cm^−1^, indicating that the EnCA moiety was successfully incorporated into the phthalocyanine framework and the disappearance of the C≡N band verified the completion of the cyclotetramerization reaction, forming the phthalocyanine macrocycle. The UV–Vis spectrum displayed intense Q-bands at 6784 and 617 nm and a B (Soret) band at 295 nm, characteristic of metal phthalocyanines for EnCA-CoPc (Figure 4). The slight splitting and bathochromic shift of Q-bands are consistent with a D_4_h-symmetric Co(II) phthalocyanine structure and the paramagnetic nature of the Co^2+^ center. The MALDI-TOF-MS peak at *m*/*z* = 1333.32 [M+H]^+^ further confirmed molecular structure of EnCA-CoPc (Figure 5A). Similarly, UV–Vis spectrum of EnCA-CuPc showed Q-bands at 682 and 615 nm and a B-band at 342 nm, all characteristic of Cu(II) phthalocyanines (Figure 4). Compared with the CoPc analog, the CuPc complex exhibited a red-shifted Q-band (682 nm) and higher molar absorptivity (log ε = 5.20), indicating stronger π–π* delocalization and enhanced visible-light absorption due to the paramagnetic Cu^2+^ center. The MALDI-TOF-MS peak at *m*/*z* = 1335.64 [M-H]^+^ further confirmed molecular structure of EnCA-CoPc (Figure 5B).

**Figure 6:**
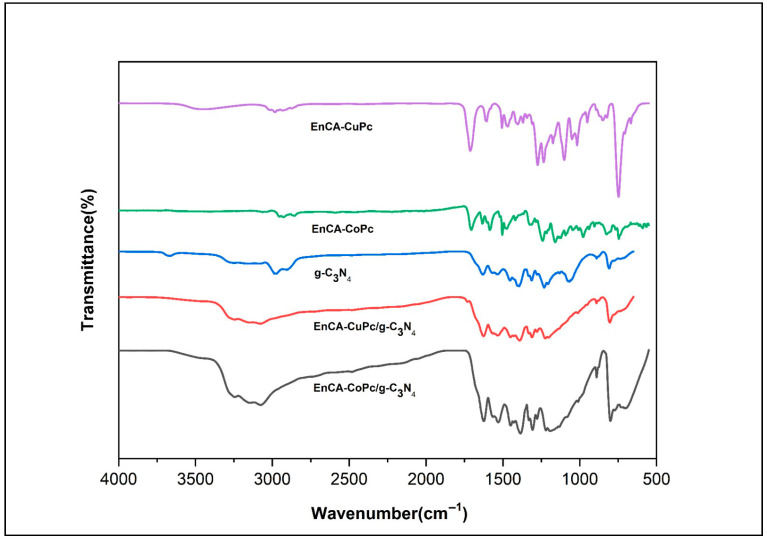
FT-IR spectra of g-C_3_N_4_, EnCA-CoPc, EnCA-CuPc, EnCA-CoPc/g-C_3_N_4_ and EnCA-CuPc/g-C_3_N_4._

**Figure 7:**
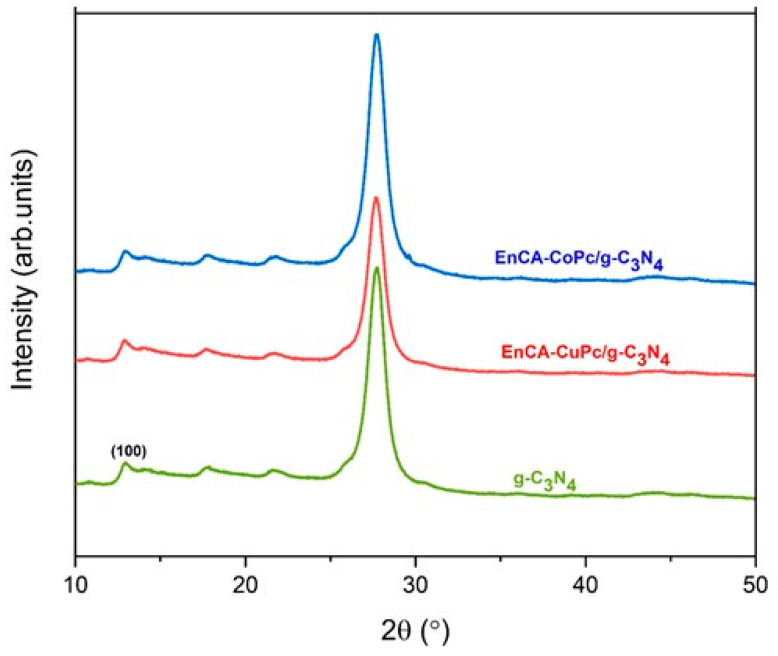
XRD spectra of EnCA-CoPc/g-C_3_N_4_ and EnCA-CuPc/g-C_3_N_4_ and g-C_3_N_4._

**Figure 8:**
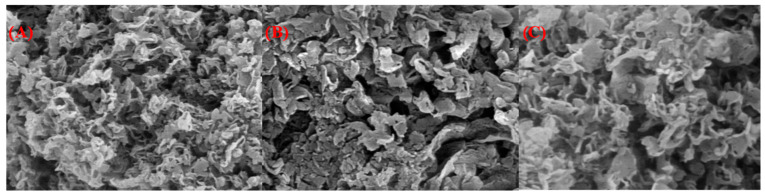
SEM images of (**A**) g-C_3_N_4_ (**B**) EnCA-CoPc/g-C_3_N_4_ (**C**) EnCA-CuPc/g-C_3_N_4._

The structural interactions between phthalocyanine (MPc) and g-C_3_N_4_ were confirmed by FT-IR, XRD and SEM analyses, these techniques provided clear evidence of π–π stacking between the two components. In the FT-IR spectra, the characteristic bands of g-C_3_N_4_ appeared at around 810 cm^−1^ (triazine ring breathing) and in the 1200–1650 cm^−1^ region (C–N and C=N stretching), indicating that the basic framework of g-C_3_N_4_ remained intact after composite formation. However, slight red shifts of the C–N/C=N vibrations and the weakening of the broad N–H stretching band (3100–3300 cm^−1^) were observed, suggesting strong π–π interactions and possible hydrogen bonding between the aromatic π-systems of MPc and g-C_3_N_4_ (Figure 6). In addition, the retention of MPc-related C=O and Ar–C–O bands confirmed the successful incorporation of the phthalocyanine moiety onto the g-C_3_N_4_ surface. XRD patterns further supported these findings: pristine g-C_3_N_4_ exhibited two major diffraction peaks at 13.1° and 27.4° corresponding to in-plane structural packing and interlayer stacking of the conjugated layers. After MPc loading, the peak slightly shifted toward lower angles (≈27.0°) and decreased in intensity, indicating an increased interlayer spacing due to MPc insertion and a partial disturbance of the ordered stacking structure. The reflection also broadened slightly, suggesting reduced in-plane crystallinity caused by MPc–g-C_3_N_4_ interaction. Together, FT-IR and XRD results confirm that MPc molecules are effectively anchored onto g-C_3_N_4_ through noncovalent π–π stacking interactions, enhancing charge transfer pathways and creating a stable hybrid interface beneficial for photocatalytic activity (Figure 7). Figure 8A–C show the images of g-C_3_N_4_, EnCA-CoPc/g-C_3_N_4_, EnCA-CuPc/g-C_3_N_4_ to prove noncovalent π–π stacking interactions between metallophthalocyanines and g-C_3_N_4_.

### 2.2. Photocatalytical Studies

**Figure 9:**
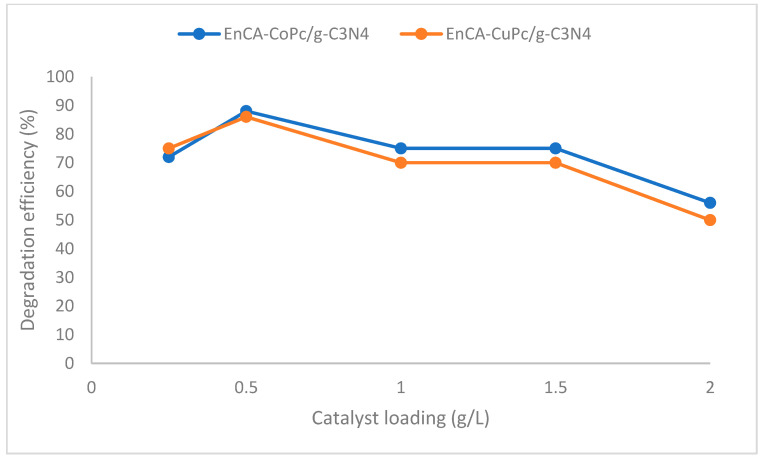
Effect of catalyst loading on the photocatalytic performance of EnCA-CoPc/g-C_3_N_4_ and EnCA-CuPc/g-C_3_N_4_.

The photocatalytic efficiency of MPc/g-C_3_N_4_ composites in the degradation of chlorophenolic compounds is critically dependent on catalyst loading, which directly modulates the density of accessible active sites and the photonic absorption capacity of the system. At suboptimal loadings, the limited availability of catalytic sites constrains the generation of photogenerated electron–hole pairs, thereby attenuating the formation of key reactive oxygen species, including hydroxyl (•OH) and superoxide (O_2_^•−^) radicals, and consequently impeding the degradation kinetics of the target pollutants. Conversely, optimal catalyst loadings can induce light scattering and shielding phenomena, wherein the upper layers of the photocatalyst attenuate incident irradiation, reducing photon penetration into deeper layers and diminishing the overall photocatalytic activity. Moreover, excessive catalyst loading can promote the agglomeration of MPc/g-C_3_N_4_ particles, thereby diminishing the effective surface area available for pollutant adsorption and reactive radical generation. Optimal catalyst loading represents a balance between these competing effects, providing sufficient surface area, enhanced light-harvesting efficiency, and efficient generation of reactive oxygen species, ultimately maximizing photocatalytic degradation rates. Previous studies on MPc/g-C_3_N_4_ systems report optimum loadings typically within the range of 0.25–2.0 g/L; however, the precise value is contingent upon the specific MPc species, the morphology of g-C_3_N_4_, light intensity, and the concentration of the target pollutant [22,23,24]. The degradation efficiency was also evaluated at a lower dosage of 0.25 g/L; however, the results indicated a decline in performance (approx. 72–75%), confirming that 0.5 g/L is the optimal loading for the current system. In the present study, catalyst loadings within the 0.25–2.0 g/L range were investigated (Figure 9), and the optimal loading was identified as 0.5 g/L for both photocatalysts, corresponding to 25 mg of phthalocyanine dispersed in 50 mL of ethanol.

**Figure 10:**
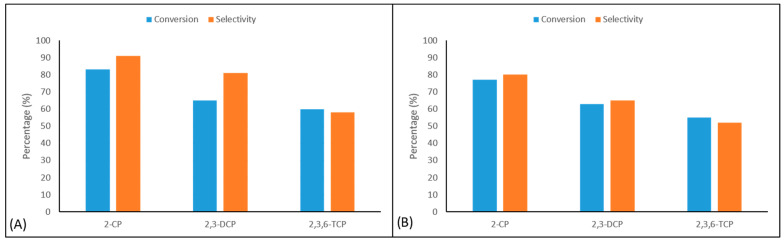
Photocatalytic selectivity and conversion using (**A**) EnCA-CoPc/g-C_3_N_4_ (**B**) EnCA-CuPc/g-C_3_N_4_ for chlorophenol derivatives. [The selectivity was calculated using the formula S = [C_product_/(C_0_ − Ct)] × 100, where C_product_ represents the concentration of the identified oxidation products and (C_0_ − Ct) is the total amount of chlorophenol converted].

**Figure 11:**
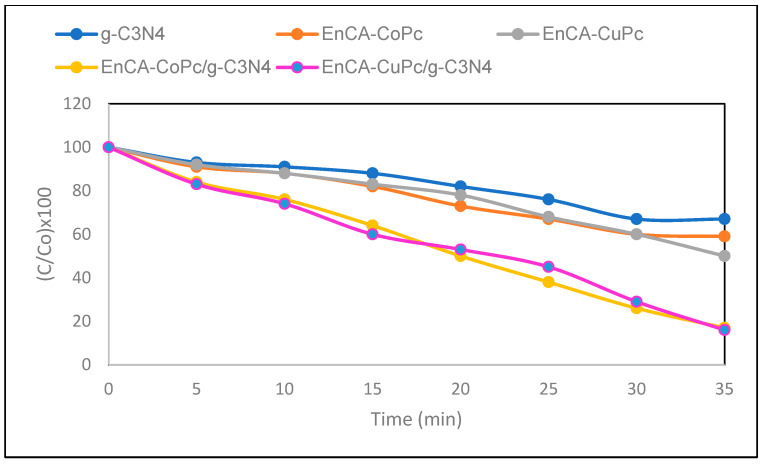
Time-dependent photocatalytic degradation (C/C_0_ × 100) of chlorophenols over different catalysts from this work.

The susceptibility of phenolic derivatives to oxidative degradation is strongly influenced by the presence of electron-withdrawing or electron-donating substituents on the aromatic ring. In this study, the oxidation of 2-chlorophenol, 2,3-dichlorophenol, and 2,3,6-trichlorophenol yielded benzoquinone derivatives as the primary products (Table 1). The electronegative chlorine atom exerts an inductive electron-withdrawing effect on the aromatic system, which intensifies with an increasing number of chlorine substituents. Conversely, chlorine can partially contribute electron density through resonance (+M mesomeric effect), predominantly at ortho and para positions. Consequently, an increase in the number of chlorines generally hinders the oxidation process. Nevertheless, it is noteworthy that the presence of chlorine atoms at ortho positions can facilitate oxidation by enabling the transfer of π-electron density within the ring. In this study, both catalysts catalyzed 2-chlorophenol with high selectivity and product conversions, and the oxidation resulted in the formation of 2-chloro-1,4-benzoquinone. The order of highest product conversions for the pollutants was as follows; 2-chlorophenol > 2,3-dichlorophenol > 2,3,6-trichlorophenol. Looking at Table 1, the highest product selectivity and conversion rate was obtained in the oxidation of 2-chlorophenol with the EnCA-CoPc/g-C_3_N_4_ catalyst (82.6% selectivity, 90.8% conversion). The lowest product selectivity was determined as 51.6% in the oxidation of 2,3,6-trichlorophenol with the EnCA-CuPc/g-C_3_N_4_ catalyst (Figure 10). CoPc systems are known from previous studies to generally yield slightly higher selectivity than CuPc [25,26,27,28,29,30] because the redox cycle (Co^2+^/Co^3+^) of the Co center is more efficient in electron transfer. The results in Table 1 also demonstrate the importance of light, the catalyst, and g-C_3_N_4_ in the degradation of pollutants. One of our objectives in this study is to use photocatalysts to degrade harmful or persistent chlorophenols into less toxic and more environmentally friendly products under mild conditions. These pollutants can generally be efficiently oxidized or reduced using photocatalysts. It was calculated that the EnCA-CoPc/g-C_3_N_4_ catalyst degraded 2-chlorophenol by 82.6%, while the EnCA-CuPc/g-C_3_N_4_ catalyst degraded 2-chlorophenol by 77.4% (Figure 11).

In light of the existing literature, the photocatalytic oxidation of chlorophenols—including 2-chlorophenol (2-CP), 2,3-dichlorophenol (2,3-DCP), and 2,3,6-trichlorophenol (2,3,6-TCP)—is understood to proceed predominantly through a radical-mediated pathway under irradiation in the presence of photocatalysts such as TiO_2_, g-C_3_N_4_, or phthalocyanine-based systems [6,22,31]. Upon absorption of photons with energy equal to or greater than the semiconductor bandgap, the photocatalyst generates electron–hole pairs (e^−^/h^+^), promoting electrons from the valence band (VB) to the conduction band (CB):

Catalyst + hν → e^−^ (CB) + h^+^ (VB)

The photogenerated holes and electrons subsequently migrate to the catalyst surface, where they participate in oxidation and reduction reactions, respectively. The VB holes oxidize surface-bound water or hydroxyl groups to form hydroxyl radicals (•OH)—highly potent, nonselective oxidizing agents:

h^+^ + H_2_O → •OH + H^+^

Meanwhile, CB electrons are captured by molecular oxygen adsorbed on the catalyst surface, producing superoxide radicals (O_2_^•−^):

e^−^ + O_2_ → O_2_^•−^

**Figure 12:**
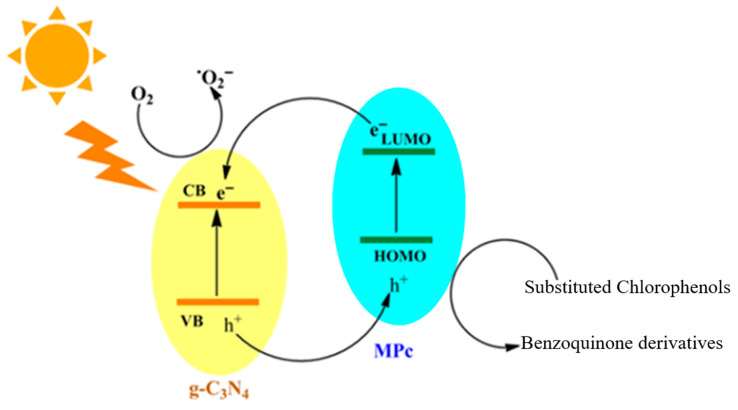
Proposed mechanism for the photocatalytic degradation of substitute chlorophenols under light using MPc/g-C_3_N_4_ composite.

Hydroxyl radicals (•OH) possess sufficient redox potential to oxidize aromatic pollutants through electrophilic attack, while superoxide radicals (O_2_^•−^) promote stepwise electron transfer and partially oxidizing transformations [32,33,34] (Figure 12). Together with direct hole oxidation, these species establish a multiroute oxidative environment. Highly reactive hydroxyl (•OH) and superoxide (O_2_^•−^) radicals initiate the degradation of chlorophenols through electrophilic attack on the aromatic ring, leading first to hydroxylation and the formation of dihydroxy intermediates such as catechol- and hydroquinone-type compounds [35,36,37]. Consecutive hydroxylation steps weaken the C–Cl bonds, resulting in progressive dechlorination and the subsequent oxidation of these intermediates to benzoquinone derivatives [38,39,40].

**Figure 13:**
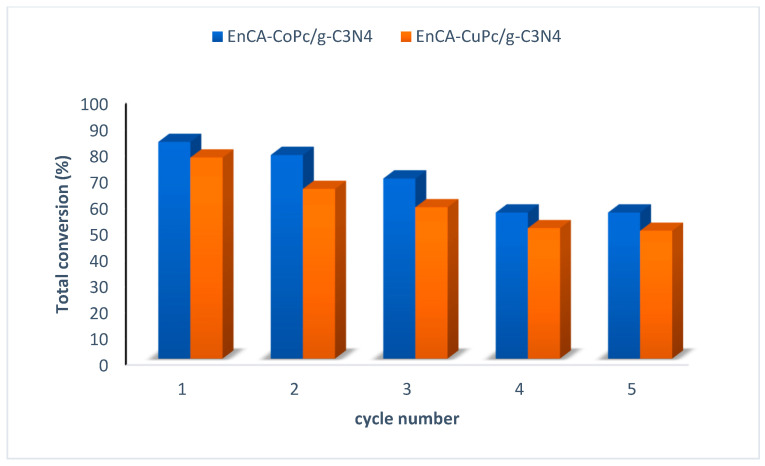
Recycling studies of En-CA-CoPc/g-C_3_N_4_ and EnCA-CuPc/g-C_3_N_4._

Photocatalyst recyclability is a critical consideration for both environmental sustainability and economic viability. Given that photocatalysts often incorporate costly metal centers or complex composite architectures, their reuse across multiple reaction cycles can substantially reduce operational costs. Moreover, preserving the structural integrity and photochemical activity of the catalyst enhances the reliability and longevity of the photocatalytic system. In the present study, recycling experiments were conducted to evaluate the durability and reusability of the employed photocatalysts, specifically assessing potential performance loss upon successive reuse. The catalysts were recovered after each reaction cycle, washed, and reintroduced under identical reaction conditions to monitor changes in degradation efficiency and product selectivity, providing insights into their operational stability over multiple cycles. In this study, both photocatalysts retained over 50% total product conversion after five successive reaction cycles, indicating significant preservation of their catalytic activity and structural integrity. Notably, the recycling experiments revealed that the EnCA-CoPc/g-C_3_N_4_ catalyst maintained approximately 68% total product conversion after five cycles, whereas the EnCA-CuPc/g-C_3_N_4_ catalyst exhibited approximately 60% total product conversion after four cycles. These results underscore the considerable durability and reusability of the synthesized catalysts under repeated operational conditions, highlighting their potential for sustainable photocatalytic applications (Figure 13).

In recent years, Advanced Oxidation Processes (AOPs) have emerged as a paramount strategy for the degradation of recalcitrant organic contaminants, owing to their ability to generate highly reactive species that facilitate complete mineralization. To enhance the efficiency of these processes, a diverse array of functional materials has been extensively investigated for their photocatalytic and catalytic activities [41,42,43]. Within this framework, the development of synergistic heterojunctions has gained significant traction as a means to overcome the limitations of single-component catalysts. Consequently, over the past decade, numerous studies have reported the preparation of phthalocyanine (MPc) and graphitic carbon nitride (g-C_3_N_4_) hybrid materials and investigated their applications across diverse fields [44,45,46,47,48,49,50,51,52,53]. However, only a limited number of studies have specifically focused on the photocatalytic degradation of phenolic or dye pollutants [54,55,56]. A summary of representative investigations involving ZnPc, CoPc, FePc, and NiPc incorporated into g-C_3_N_4_ matrices is presented in Table 2 [57,58,59,60,61]. Zada et al. demonstrated that ZnPc/g-C_3_N_4_ catalyzed the degradation of 2,4-dichlorophenol (2,4-DCP), achieving 85–90% conversion within 120 min under visible light irradiation [57], whereas Wang et al. reported approximately 80% degradation of 2,4-DCP in just 90 min in the presence of CoPc/g-C_3_N_4_ [58], highlighting the significant influence of redox-active metal centers on catalytic performance. Li and co-workers further showed that incorporating g-C_3_N_4_ into a FePc/CeO_2_ heterojunction led to ~90% degradation of 2,4-DCP within 60 min [59], whereas Khan et al. observed 80% removal of 4-chlorophenol using NiPc/g-C_3_N_4_ under similar conditions [60]. In comparison, photocatalytic degradation with pristine g-C_3_N_4_ typically achieves only 45–60% conversion for 2,4-DCP and 4-chlorophenol [61], emphasizing the critical role of phthalocyanines in enhancing photocatalytic efficiency. The incorporation of MPc units not only extends visible light absorption but also facilitates effective electron–hole separation, thereby promoting the generation of reactive species such as superoxide radicals (O_2_^•−^), photogenerated holes (h^+^), and, in some cases, singlet oxygen (^1^O_2_), which drive oxidative degradation. Despite these advances, a critical analysis of the literature (Table 2) reveals that studies addressing highly chlorinated phenolic compounds remain scarce, and essential information on photocatalyst photostability, recyclability, and by-product identification is often lacking, representing significant gaps in the field.

**Figure 14:**
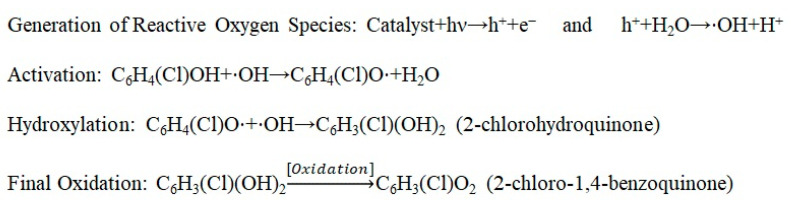
A plausible mechanism for the photocatalytic oxidation of 2-chlorophenol into 2-chloro-1,4-benzoquinone via radical intermediates.

The transformation begins with the photocatalytic activation of the catalyst (such as TiO_2_), where incident light provides enough energy to promote electrons to the conduction band, leaving behind highly reactive “holes” (h^+^) in the valence band. These holes oxidize water or adsorbed hydroxyl ions to generate hydroxyl radicals (⋅OH), which act as the primary oxidants. The mechanism proceeds via the abstraction of a hydrogen atom from the phenolic ^−^OH group of 2-chlorophenol, creating a 2-chlorophenoxy radical (Figure 14). Due to the resonance stabilization of this radical, the electron density shifts significantly to the para-position (carbon 4), making it the most favorable site for a second hydroxyl radical attack. This nucleophilic-like addition forms 2-chlorohydroquinone as a critical intermediate. In the final stage, the intermediate undergoes a rapid two-step dehydrogenation (oxidation), involving the loss of two protons and two electrons—often facilitated by the catalyst surface or dissolved oxygen—to yield the stable conjugated system of 2-chloro-1,4-benzoquinone [62,63,64].

## 3. Experimental

### 3.1. Materials

All materials, equipment, general procedures of EnCA-CN, EnCA-CoPc, EnCA-CuPc, EnCA-CoPc/g-C_3_N_4_, EnCA-CuPc/g-C_3_N_4_ and photocatalytic procedure are given as Supplementary File.

### 3.2. Synthesis

#### 3.2.1. Synthesis of Ethyl (2Z)-3-(4-(2,3-Dicyanophenoxy)phenyl)acrylate (EnpCA-CN)

Yield: 2.64 g (80%); FT-IR: υmax /cm^−1^ 3078 (Ar–H), 2987 (Alif. C–H), 2225 (C≡N), 1703 (C=O), 1571, 1444, 1270, 1160 (ArC–O), 980, 841. ^1^H-NMR. (DMSO), (d:ppm): 8.13 (d, *J*: 8.8, H, Ar-H), 7.85 (d, *J*: 8.8, 3H, Ar-H), 7.80 (s, H, Ar-H), 7.74 (d, *J*: 8.0, H, Ar-H), 7.52 (d, *J*: 6.0, 2H, CH=CH), 6.62 (d, *J*: 8.4, H, Ar-H), 4.23–4.17 (q, *J*: 7.2, 2H, CH2-O), 1.25 (t, *J*: 6.8, 3H, CH3). ^13^C-NMR. (DMSO), (d:ppm): 166.62, 163.56, 156.12, 143.72, 136.83, 131.90, 131.20, 123.81, 123.86, 123.12, 118.68, 117.27, 116.32, 115.32, 109.27, 60.54, 14.67. MALDI-TOF-MS: *m*/*z*: calcd. 318.32; found 318.310 [M]^+^, 303.260 [M-CH_3_]^+^, 283.652 [M-C_2_H_5_-6H]^+^.

#### 3.2.2. Co(II) Phthalocyanine (EnCA-CoPc)

The green solid product had good solubility in THF, DMF, DMSO and CHCl_3_ solvents. Yield: 280 mg (%45); FT-IR: υmax /cm^−1^ 3035 (Ar–H), 2954–2867 (Alif. C–H), 1705 (C=O), 1585, 1503, 1474, 1420, 1323, 1241–1159(ArC–O) 1091, 1046,1007, 939, 905, 825,824, 745. UV/Vis (DMSO): λmax (log ε) 684 (5.26), 617 (4.66), 295 (5.38). MALDI-TOF-MS: *m*/*z*: calcd. 1332.17; found 1333.32 [M+H]^+^.

#### 3.2.3. Cu(II) Phthalocyanine (EnCA-CuPc)

The green solid product had good solubility in THF, DMF, DMSO, and CHCl_3_ solvents. Yield: 300 mg (%50); FT-IR: υmax /cm^−1^ 3016 (Ar–H), 2982–2870 (Alif. C–H), 1712 (C=O), 1506, 1467, 1403, 1368, 1343, 1273–1174 (ArC–O), 1049, 1017, 950, 850, 822, 746. UV/Vis (DMSO): λmax (log ε) 682 (5.20), 615 (4.64), 342 (4.85). MALDI-TOF-MS *m*/*z*: calcd. 1336.78; found 1335.64 [M-H]^+^.

## 4. Conclusions

In summary, the present study demonstrates that EnCA-CoPc/g-C_3_N_4_ and EnCA-CuPc/g-C_3_N_4_ photocatalysts exhibit remarkable photocatalytic activity and structural stability for the degradation of chlorophenolic compounds under visible light irradiation. The optimal catalyst loading was determined to be 0.5 g/L, achieving an optimal balance between the accessibility of active sites and the efficiency of light absorption. Both photocatalysts efficiently facilitated the degradation of 2-chlorophenol, 2,3-dichlorophenol, and 2,3,6-trichlorophenol via radical-mediated oxidation pathways, with benzoquinone derivatives identified as the principal intermediates. Among the tested systems, EnCA-CoPc/g-C_3_N_4_ displayed superior catalytic performance and selectivity, achieving 90.8% conversion and 82.6% selectivity for 2-chlorophenol, which can be attributed to the efficient Co^2+^/Co^3+^ redox cycle that promotes effective charge separation and transfer. Recycling studies further confirmed the durability of both photocatalysts, with over 50% of their initial activity retained after five consecutive cycles. Building upon the robust empirical foundation established in this study, future research is poised to advance the frontiers of environmental remediation through innovative catalytic strategies. A primary objective of forthcoming investigations will be to enhance the degradation kinetics of these hybrid catalysts, particularly when targeting highly chlorinated and recalcitrant organic pollutants that exhibit greater resistance to oxidative processes. By leveraging the successful performance of the current system, future work may focus on optimizing the composite architecture through advanced chemical functionalization or the exploration of diverse g-C_3_N_4_ nanostructures to maximize photocatalytic stability and maintain peak efficiency well beyond five cycles. Furthermore, evaluating these materials within complex, real-world wastewater matrices and conducting exhaustive mechanistic analyses of degradation by-products will be essential to validate the system’s environmental safety and industrial scalability. These strategic research directions will facilitate the development of sustainable, high-performance solutions for the mineralization of a broad spectrum of persistent organic pollutants.

## Figures and Tables

**Table 1:** All photocatalytic results with both catalysts for chlorophenols degradation.

Photocatalyst	Pollutant	Product ^a^	Product Selectivity ^b^ (%)	Total Conversion (%)
EnCA-CoPc/g-C_3_N_4_	2-CP	2-Chloro-1,4-BQ	90.8	82.6
EnCA-CuPc/g-C_3_N_4_	2-CP	2-Chloro-1,4-BQ	80.0	77.4
EnCA-CoPc/g-C_3_N_4_	2,3-DCP	2,3-Dichloro-1,4-BQ	81.4	65.4
EnCA-CuPc/g-C_3_N_4_	2,3-DCP	2,3-Dichloro-1,4-BQ	65.2	62.6
EnCA-CoPc/g-C_3_N_4_	2,3,6-TCP	2,3,6-Trichloro-1,4-BQ	58.5	59.4
EnCA-CuPc/g-C_3_N_4_	2,3,6-TCP	2,3,6-Trichloro-1,4-BQ	51.6	55.5
g-C_3_N_4_	2-CP, 2,3-DCP, 2,3,6-TCP	BQ derivatives	~65	≥45.0
EnCA-CoPc	2-CP, 2,3-DCP, 2,3,6-TCP	BQ derivatives	>55	≥65.0
EnCA-CuPc	2-CP, 2,3-DCP, 2,3,6-TCP	BQ derivatives	>50	≥49.0
Absence of cat.	2-CP, 2,3-DCP, 2,3,6-TCP	BQ derivatives	>25	≥23.0
Absence of light	2-CP, 2,3-DCP, 2,3,6-TCP	BQ derivatives	>12	≥15

^a^: Major product, ^b^: Selectivity of benzoquinone derivatives, pollutant concentration 1.2 × 10^−5^ M, optimum loading was determined to be 0.5 g/L for both photocatalysts (25 mg of phthalocyanine in 50 mL ethanol).

**Table 2:** A summary of the studies on ZnPc, CoPc, FePc and NiPc incorporated into g-C_3_N_4_ matrices.

Photocatalyst System	Pollutant	Light Conditions	Degradation/Conversion Performance	Dominant Reactive Species & Mechanism (Condensed)	Ref.
ZnPc ^a^/g-C_3_N_4_ nanocomposite	2,4-DCP	Visible light (λ > 420 nm)	≈85% degradation in 120 min	O_2_^•−^ and h^+^ driven oxidation; improved charge separation	[57]
CoPc ^b^/g-C_3_N_4_ composite	2,4-DCP	Visible light (λ > 400 nm)	≈78% degradation in 90 min	O_2_^•−^ and ^1^O_2_ generation; enhanced electron transfer	[58]
FePc ^c^/CeO_2_/g-C_3_N_4_	2,4-DCP	450 nm LED	≈90% in 100 min	Z-scheme charge transfer; h^+^ and O_2_^•−^ dominant	[59]
NiPc ^d^/g-C_3_N_4_ + H_2_O_2_	4-CP	Visible light (λ > 420 nm) + H_2_O_2_	≈80% in 60 min	•OH and O_2_^•−^ mediated oxidation	[60]
Bare g-C_3_N_4_^e^	2,4-DCP, 4-CP	Visible light	≈45–60%	Limited activity; h^+^ and O_2_^•−^ primary species	[61]
EnCA-CoPc/g-C_3_N_4_ (this study)	2-CP, 2,3-DCP, 2,3,6-TCP	Visible light	82.6% conversion and 90.8% selectivity (2-CP); decreasing efficiency: 2-CP > 2,3-DCP > 2,3,6-TCP; retains 68% activity after 5 cycles	O_2_^•−^ and h^+^ driven oxidation; π–π coupled MPc/g-C_3_N_4_ interface enhances charge separation; benzoquinone formation as major pathway	This work
EnCA-CuPc/g-C_3_N_4_ (this study)	2-CP, 2,3-DCP, 2,3,6-TCP	Visible light	≈90% pollutant removal; similar degradation order; retains 60% activity after 4 cycles	O_2_^•−^ dominant radical route; improved electron transfer through Cu(II) redox centers; quinone intermediates identified	This work

^a^ Zn-phthalocyanine/g-C_3_N_4_ nanocomposite, ^b^ Co-phthalocyanine/g-C_3_N_4_ composite, ^c^ Fe-phthalocyanine/g-C_3_N_4_ Z-scheme heterostructure, ^d^ Ni-phthalocyanine/g-C_3_N_4_, ^e^ non-phthalocyanine/g-C_3_N_4_. 2,4-DCP: 2,4-Dichlorophenol, 4-CP:4-Chlorophenol.

## Data Availability

The datasets used and/or analyzed during the current study are available from the corresponding author on reasonable request.

## Supplementary Materials

The following supporting information can be downloaded at: https://www.mdpi.com/article/10.3390/molecules31020213/s1.
