# Supplementary material for: Synthesis and Photocatalytic Evaluation of CoPc/g-C3N4 and CuPc/g-C3N4 Catalysts for Efficient Degradation of Chlorinated Phenols
Source: Molecules. 2026 Jan 8;31(2):213. doi: 10.3390/molecules31020213 (PMC12843753; doi:10.3390/molecules31020213)
Supplement: Supplementary file 1 [file molecules-31-00213-s001.zip › molecules-4065090-supplementary.pdf]

CuPc/g-C_3N_4 Catalysts for Efficient Degradation of Chlorinated Phenols

Cagla Akkol [1,2], Yasemin Caglar [3,*] and Ece Tugba Saka [1,*]

1 Department of Chemistry, Faculty of Science, Karadeniz Technical University, Trabzon 61080, Türkiye; akkolcagla@gmail.com
2 Department of Chemistry, Institue of Science, Karadeniz Technical University, Trabzon 61080, Türkiye
3 Department of Genetic and Bioengineering, Faculty of Engineering, Giresun University, Giresun 28200, Türkiye
* Correspondence: yasemin.caglar@giresun.edu.tr (Y.C.); esaka@ktu.edu.tr or ece_t_saka@hotmail.com (E.T.S.)

1.Materials

All reactions were carried under a dry nitrogen atmosphere using Standard Schlenk techniques. All chemicals, solvents, and reagents were of reagent grade quality and were used as purchased from commercial sources. All solvents were dried and purified as described by reported procedure [1]. 4-Nitrophthalonitrile [2] were prepared according to the literature procedure. 4nitrophenol were purchased from Sigma-Aldrich and used without further purification and chemical treatment.

2. Equipment

The IR spectra were recorded on a Perkin Elmer 1600 FT-IR spectrophotometer using KBr pellets. ^{1}H-NMR and ^{13}C-NMR spectra were recorded on a Varian Mercury 400 MHz spectrometer in CDCl3. Chemical shifts were reported relative to Me4Si as internal standard. MALDI-MS of complexes were obtained in dihydroxybenzoic acid as MALDI matrix using nitrogen laser accumulating 50 laser shots using Bruker Microflex LT MALDI-TOF mass spectrometer. Optical spectra in the UV-vis region were recorded with a Perkin Elmer Lambda 25 spectrophotometer. The identification of degradation products was carried out using an Agilent 7820A Gas Chromatograph (GC). In the absence of MS equipment, the standard addition method was employed, whereby the intermediates were identified by comparing their

retention times and peak area enhancements with those of authentic commercial standards. The morphologies of all surfaces were examined with Everhart-Thornley detector (ETD) at 20 kV by a scanning electron microscope (SEM: Thermo Scientific Apreo 2S). The existence of phases on all surfaces was investigated with a Cu-Kα radiation from 20° to 90° at a detecting speed of 1° min^{-1} by X-ray diffraction (XRD: GNR Europe 600) device.

3. Synthesis of ethyl (2Z)-3-(4-(2,3-dicyanophenoxy)phenyl)acrylate (EnpCA-CN)

Ethyl (2Z)-3-(4-hydroxyphenyl)acrylate (2 g, 6.28 mmol), 4-nitrophthalonitrile (1.1 g, 10.4 mmol), and 25 mL of dry DMF were added to a 250 mL reaction flask under a nitrogen atmosphere and stirred at 50 °C for 15 minutes. After the reaction mixture was completely dissolved, 4.51 g (32.63 mmol) of anhydrous K_2CO_3 was added to the reaction mixture in small portions over 2 hours. After removing the dissolved oxygen from the reaction medium, the reaction was continued under stirring at 50 °C for 96 hours under a nitrogen atmosphere. At the end of this period, the reaction mixture was cooled to room temperature and poured onto 100 g of ice, stirred at room temperature for 2 hours, and filtered through a crucible. The crude product formed was crystallized from ethanol. The solid product obtained was washed with cold ethyl alcohol, filtered, and dried in a vacuum desiccator. The pale yellow solid ethyl (E)-3-(4-(2,3dicyanophenoxy)phenyl)acrylate was obtained.

4. Co(II) phthalocyanine (EnCA-CoPc)

Ethyl (2Z)-3-(4-(3,4-dicyanophenoxy)phenyl)acrylate (485 mg, 3.36 mmol), 6 mL of npentanol, 8-10 drops of DBU, and anhydrous metal salt ($CoCl_2$ (225,8 mg, 0.68 mmol)) were added to a Schlenk tube and stirred at 160 °C under a nitrogen atmosphere for 12 hours. The

reaction mixture was cooled to room temperature, and 30 mL of ethanol was added to the green solution and stirred at room temperature for 1 hour. The precipitated green crude product was filtered from a crucible and dried in a vacuum desiccator. The solid product was separated in a column loaded with basic alumina. It was purified using column chromatography with $CHCl_3$:C_2H_5OH (50:1.5 v/v) solvent system. The appropriate fractions were determined by thin-layer chromatography and combined. The combined fractions were evaporated to dryness under reduced pressure in the evaporator. The green product precipitated with the addition of ethyl alcohol was filtered, washed with diethyl ether, and dried in a vacuum desiccator.

5. Cu(II) phthalocyanine (EnCA-CuPc)

Ethyl (2Z)-3-(4-(3,4-dicyanophenoxy)phenyl)acrylate (485 mg, 3.36 mmol), 6 mL of npentanol, 8-10 drops of DBU, and $CuCl_2$ (217 mg, 0.81 mmol) were added to a Schlenk tube and stirred at 160 °C under a nitrogen atmosphere for 12 hours. The reaction mixture was cooled to room temperature, and 30 mL of ethanol was added to the green solution and stirred at room temperature for 1 hour. The precipitated green crude product was filtered from a crucible and dried in a vacuum desiccator. The solid product was separated in a column loaded with basic alumina. It was purified using column chromatography with $CHCl_3$:C_2H_5OH (50:1.5 v/v) solvent system. The appropriate fractions were determined by thin-layer chromatography and combined. The combined fractions were evaporated to dryness under reduced pressure in the evaporator. The green product precipitated with the addition of ethyl alcohol was filtered, washed with diethyl ether, and dried in a vacuum desiccator.

6. Preparation of g-C_3N_4

50 g of urea is weighed. The urea is ground briefly in a mortar to make it more homogeneous. It is transferred to a porcelain crucible. The furnace is preheated. The crucible containing the urea is placed in the furnace in an air environment. It is heated at a rate of 5°C/min from 25°C

to 550°C. It is held at 550°C for 4 hours. The furnace is then turned off, and the sample is allowed to cool naturally to room temperature. Graphitic carbon nitride is a pale yellow, lightweight, and porous solid.

7. Preparation of MPc/ g-C_3N_4 [M:Co(II) or Cu(II)]

Metallophthalocyanine (25 mg) was dissolved in 50 ml of ethanol, after which g−C_3N_4 (100 mg) was added and stirred with a magnetic stirrer for 30 minutes. The mixture was then treated in an ultrasonic bath for 10 minutes to ensure a homogeneous distribution. The resulting suspension was filtered and washed several times with deionized water to remove any unanchored species or impurities. Finally, the obtained product was dried overnight in an oven at 80 °C. By loading MPc with different masses (25, 50, 75, and 100 mg), four types of MPc/g−C_3N_4 [M: Co(II) or Cu(II)] were prepared and denoted as x%MPc/g−C_3N_4 (x = 2.5, 5.0, 7.5, and 10). The average yield for the synthesized catalysts was approximately 85% based on the total mass of the precursors.

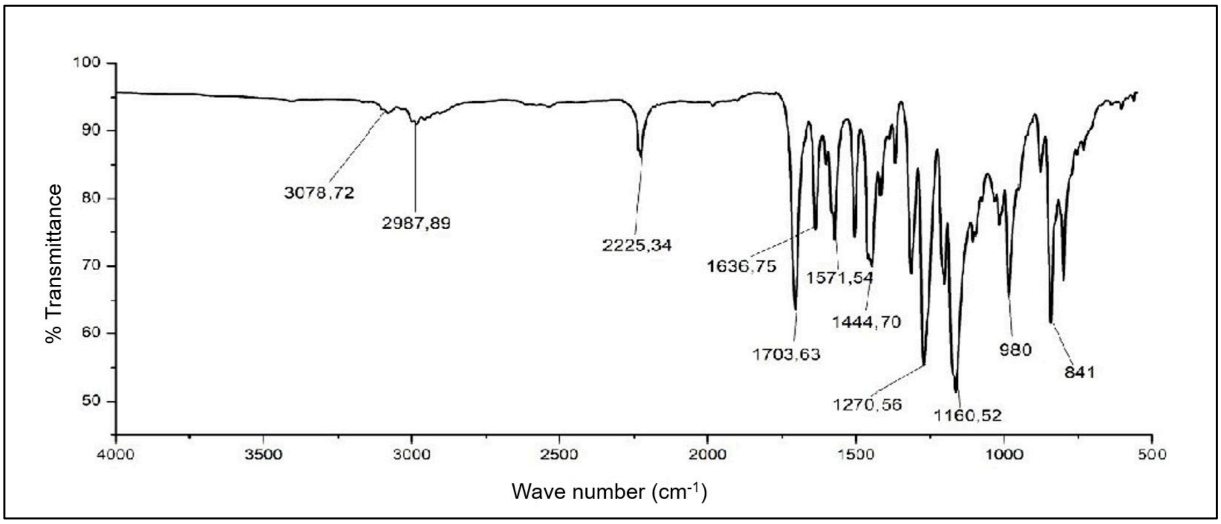

Supplementary Figure S1. FT-IR spectrum of EnCA-CN

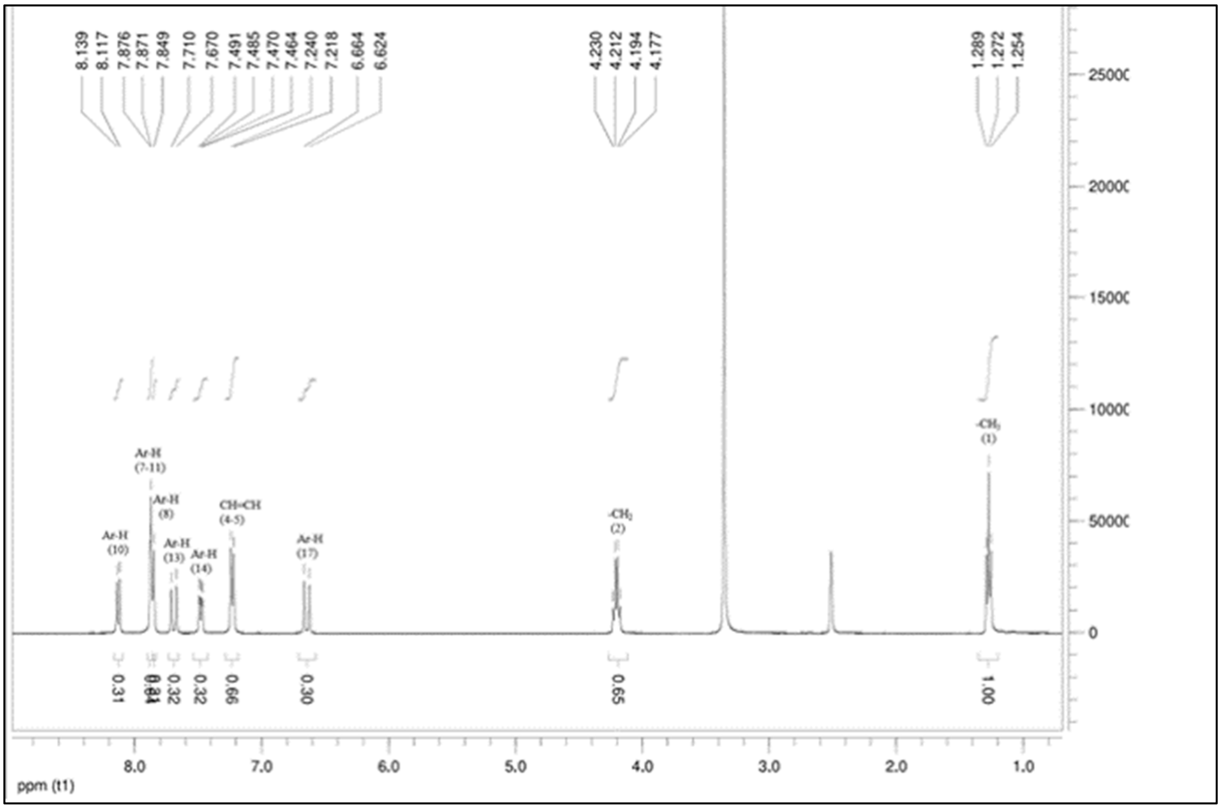

Supplementary Figure S2. ^{1}H-NMR spectrum of EnCA-CN

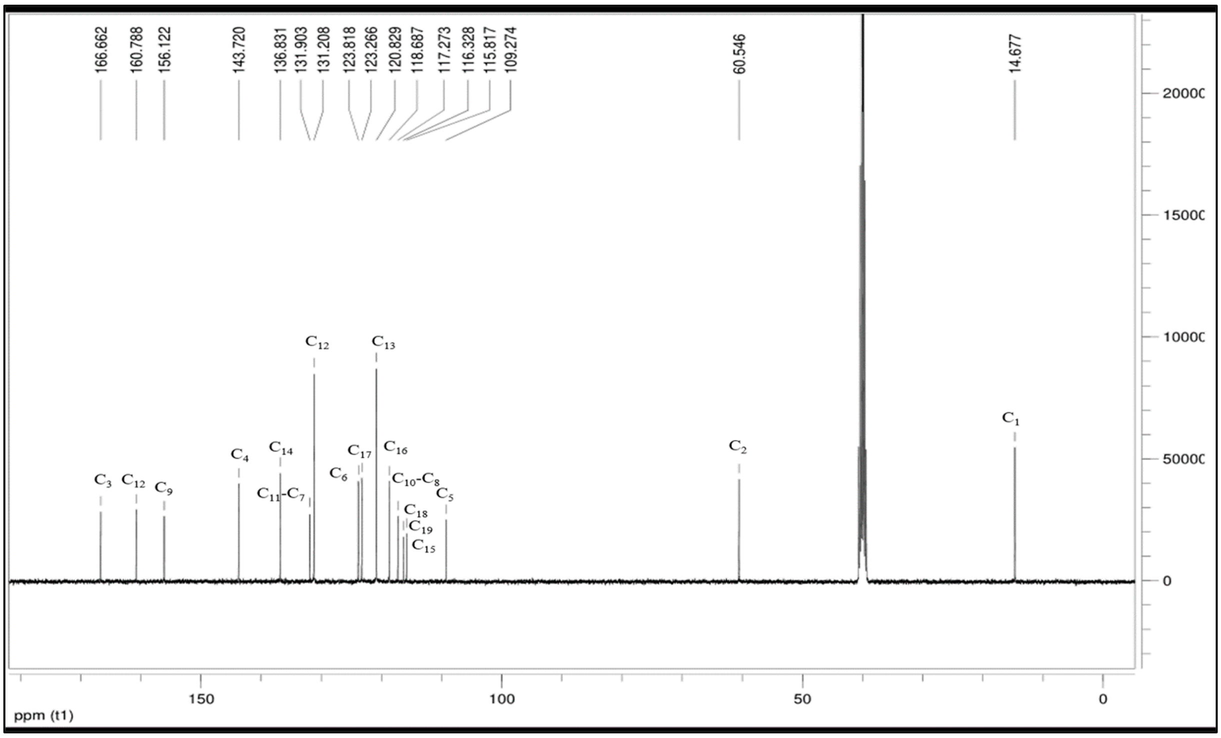

Supplementary Figure S3. ^{13}C-NMR spectrum of EnCA-CN
